# Supplementary material for: Metagenomic Analysis Reveals the Anti-Inflammatory Properties of Mare Milk
Source: Int J Mol Sci. 2025 Aug 25;26(17):8239. doi: 10.3390/ijms26178239 (PMC12428327; doi:10.3390/ijms26178239)
Supplement: Supplementary file 1 [file ijms-26-08239-s001.zip › ijms-3697638-supplementary.pdf]

Table S1. Sequencing results.

Sample	Raw Data （M）	Raw Reads	Clean Data （M）	Clean Q20	Clean Q30	Clean GC(%)	Effective(%)
DW1	6284.03	41893530	6280.39	98.01	94.27	48.34	99.942
DW2	6816.63	45444186	6811.44	97.83	93.97	47.13	99.924
DW3	6773.58	45157210	6769.15	97.16	92.41	48.61	99.935
DW4	6639.70	44264674	6635.68	97.84	93.84	49.69	99.939
DW5	6561.48	43743208	6557.39	97.53	93.19	50.23	99.938
DW6	6209.20	41394696	6201.42	97.86	94	48.94	99.875
DW7	5992.54	39950234	5990.17	97.94	94.1	49.97	99.961
DW8	6205.35	41369028	6201.41	98.06	94.45	49.64	99.936
MM1	6432.26	42881744	6427.69	97.69	93.5	49.97	99.929
MM2	6244.38	41629172	6240.65	97.97	94.17	49.47	99.94
MM3	6263.09	41753952	6257.84	97.84	93.83	49.14	99.916
MM4	6596.48	43976546	6592.88	97.67	93.47	48.4	99.945
MM5	6543.04	43620258	6539.07	97.48	93.04	49.88	99.939
MM6	6660.45	44403008	6655.64	97.62	93.39	48.79	99.928
MM7	5984.32	39895498	5981.46	97.59	93.34	49.2	99.952
MM8	5895.50	39303324	5888.28	97.55	93.27	46.41	99.878
K1	6360.37	42402460	6356.26	97.42	92.99	49.66	99.935
K2	6455.78	43038530	6452.08	97.85	93.97	50.11	99.943
K3	6383.64	42557604	6378.52	97.52	93.18	47.78	99.92
K4	6387.72	42584804	6384.69	97.64	93.45	48.42	99.952
K5	6332.12	42214162	6329.32	97.6	93.44	49.75	99.956
K6	6374.37	42495812	6369.61	97.59	93.5	46.98	99.925
K7	6571.53	43810226	6568.60	97.2	92.41	48.83	99.955
K8	6347.08	42313862	6344.29	97.65	93.47	48.81	99.956
PK1	6486.95	43246340	6482.53	97.62	93.55	51.61	99.932
PK2	6320.34	42135620	6318.20	97.41	92.95	49.99	99.966
PK3	6028.96	40193060	6026.09	97.9	93.94	48.16	99.952
PK4	6725.12	44834116	6721.43	97.07	92.21	49.3	99.945

PK5	6254.45	41696364	6250.82	98	94.22	48.87	99.942
PK6	6332.36	42215722	6329.02	97.64	93.4	49.3	99.947
PK7	6032.81	40218748	6030.30	97.28	92.72	51.55	99.958
PK8	6741.47	44943138	6739.02	97.95	94.16	50.42	99.964
